# Limits of Optimization

**DOI:** 10.1007/s11023-023-09633-1

**Published:** 2023-04-06

**Authors:** Cesare Carissimo, Marcin Korecki

**Affiliations:** Computational Social Science, ETH Zurich, Stampfenbachstrasse 48, 8006 Zurich, Switzerland

**Keywords:** Optimization, Artificial intelligence, Complex systems, Ethics, Epistemology, Philosophy of science

## Abstract

Optimization is about finding the best available object with respect to an objective function. Mathematics and quantitative sciences have been highly successful in formulating problems as optimization problems, and constructing clever processes that find optimal objects from sets of objects. As computers have become readily available to most people, optimization and optimized processes play a very broad role in societies. It is not obvious, however, that the optimization processes that work for mathematics and abstract objects should be readily applied to complex and open social systems. In this paper we set forth a framework to understand when optimization is limited, particularly for complex and open social systems.

## Introduction

A pernicious quality of thought pervades the engineering sciences. Automation, machine learning, artificial intelligence, economics, and control comprise a non-exhaustive list of fields dominated by the perspective that: challenges are symptoms to be cured with optimization (Intriligator, 2002; Lewis et al., 2012; Passino, 2005). When optimization fails it is often believed that size is the limiting factor; that a bigger, more powerful optimization method would surely suffice (Schwartz et al., 2020). From this perspective, the right technological progress may eventually cure all symptoms. There is no proof, however, that this is the case. Quite the contrary, there are good reasons to believe that optimization is limited by its own necessities.

The social sciences have not been immune to this quality of thought. Computational advances allow data-mining and large scale simulations of human behaviour, which promise knowledge and understanding while concealing their inadequacies (Shmueli, 2017). Optimization is used both to construct optimal models (LeCun et al., 2015) and find the optimal configuration of a modelled phenomenon (Lewis et al., 2012). When a quantitative model of a phenomenon is constructed, it can always be used to try to optimize the phenomenon. We argue that doing this for open and complex systems can lead to undesirable outcomes.

We point to the *is-ought* problem in philosophy, that what *ought* to be cannot be derived from what *is*. A model may be used to understand the complexity of a system and plausibly reduce what *is* into simpler and defined components. A model can also be used to determine what might be, given different circumstances and settings for the defined components, but it would then be a fallacy to conclude what *ought* to be from the model results, for the system being modeled.

It is the case, however, that social systems can and are being modeled. Though these models may not be perfect, and decreasingly so for systems of greater complexity, it is still the case that optimization can and is being used to determine settings of these models that are ordered above other settings in terms of quantifiable metrics. In such situations, if optimizations are interpreted as determining settings that are ‘right’ from settings that are ‘wrong’, from a philosophical perspective, the optimization can be considered, perhaps provokingly, a form of *quantitative ‘ethics’*. Conversely ethics may be framed as a *‘qualitative optimization’*, where the ethical judgment of goodness or badness rests on the moral qualities of the object. This term emphasises the qualitative dimension that might be present in ethics and the tension with the purely quantitative process of optimisation. On another level of abstraction both optimization and ethics raise a multitude of axiological questions pertaining to how the value of an object can be established. We do not wish to take a fixed position on an ontic difference between quantities and qualia (which can also be seen as related to the debate on computationalist perspectives which we will expound on later, and relate to our arguments from both sides). We rather wish to point out that, optimization can and has been used to provide answers to ethical quandaries, and wish to accentuate this link before we present some limits of optimization. Such a perspective would only encapsulate some of the characteristics of what we understand as philosophical ethics (hence the quotation marks). It is worth noting that a view that explicitly links optimisation with ethics exists in the form of some applied ethics, for instance utilitarianism (where the total ‘goodness’ is to be maximized). This link can also be further explored through the lens of reductionism, by asking if ethics, taken from a philosophical perspective, could be reduced to *quantitative ‘ethics’* or optimisation: can the problems of axiology be addressed from a purely quantitative perspective or is this unquestioned reign of quantity driving us into dangerous territories?

Such questions are pertinent as computation is ubiquitous, computational resources more powerful, and artificial intelligence is on the tip of all tongues. This is because algorithms that optimize and promise to find the *best* solutions can easily lull users into a false sense of security. To begin to understand why this is the case consider the following question: *how can we find something that we do not know we are looking for?*^1^

In this paper we lay forth our arguments for the limits of optimization. We begin by defining optimization and posing the main limits (Sect. 2). We expand on each limit in separate sections: Object Limit (Sect. 3), Objective Limit (Sect. 4), and Process Limit (Sect. 5), and present an array of examples to support our claims. In an attempt to leave the reader with more questions than answers we conclude with a discussion (Sect. 6), where we pose some open questions stemming from the delineated limitations. The goal of this paper is to bring attention to the inadequacies of applying optimization uncritically. We hope to spark a multidisciplinary discussion involving fields of philosophy, social, computational and mathematical sciences.

## What is Optimization?

### Definition 1

Optimization is a process of choosing $x \in M$ such that $\forall _{m \in M} f(x) \ge f(m)$, where *M* is a set of objects and *f* is a total order on *M*.

From this we have that the *optimal* *m* is such that $\forall _{m \in M} f(x) \ge f(m)$. The set *M* represents a model, a space containing all possible objects *m* of a model—the objects *m* are particular groundings of a model, its concrete states. The total order *f* quantifies and orders the objects such that optimal objects can be established (and is a reflexive, transitive, anti-symmetric and strongly connected relation, such that all pairs of elements in *M* are comparable (Birkhoff, 1940)).

In words: optimization is the process of choosing an object from a set of objects such that it scores highest on an objective function when compared to all the other objects. Our definition of optimization captures broadly all mathematical optimizations.

### Additional Definitions

*Objective* as pertaining to the objective functions *f*, and not to be understood in terms of non-subjectivity of the matter. The objectives may not exist on their own, but are rather ‘objectivized’ by the user, in so far as the user may bring an objective into existence by pursuing it. Nonetheless, we follow the nomenclature from the field of mathematical optimization when referring to the objective.

*Phenomenon* An empirical system with behaviours observable in the real world, which one may seek to model.

*Model* A representation of a phenomenon using mathematical objects amenable to quantification.

*Quantitative (vs Qualitative)* a quantitative description relates objects to a common dimension—a quantitative difference can be measured precisely. Qualitative description of objects creates orthogonal dimensions, which might not be easily relatable to one another.

*Accuracy* A quantification of the difference of a model and the empirical phenomenon it is modelling. A perfectly accurate model will be no different than the empirical phenomenon. Such a model could be simpler than the phenomenon itself if the phenomenon is reducible—if a simpler representation suffices to capture the full breadth of the phenomenon. Note that the notion of accuracy rests on the assumption that the phenomenon is quantifiable and further that the relationship between the model and the phenomenon is also quantifiable, which may not hold true for all phenomena.

**Fig. 1:**
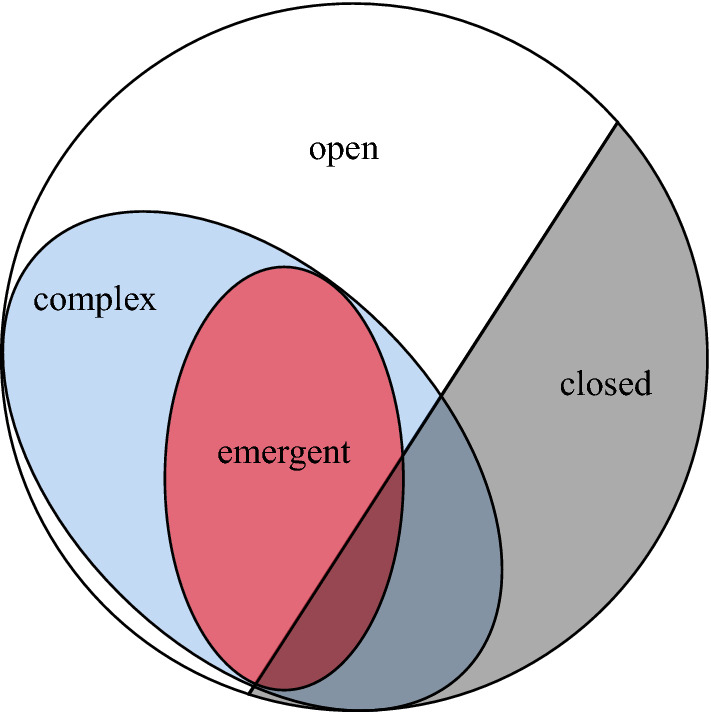
The diagram represents ‘common sense’ inclusions. Openness, complexity, and emergence are loosely defined concepts often used to define each other. Many systems found in nature are open systems. Closed systems are usually abstract models. These are best interpreted as epistemic categories, rather than ontological categories, as the latter is subject most readily to critique through computationalist perspectives

*Complex*: A system with many parts that interact non-linearly which lead to emergent behaviours which cannot be understood by studying the parts in isolation (Batty & Torrens, 2001) (see Figs. 1 and 3).

*Computationalism*^2^ Universality of computation and computer like processes, the universe and all of its contents as a machine, everything as information and information processing, sequences of inputs of finite symbolic elements manipulated by symbolic processing rules so as to yield an output; the computationalist perspective believes all phenomena to be indistinguishable from computer programs, which would seem to imply that for any phenomenon there exists a perfectly accurate model.

*Emergence* System behaviour which is ‘more than the sum of parts’. Weakly emergent behaviour arises from the interactions of many parts. Strongly emergent beahviour, on the other hand, may not be reducible to interactions of parts, as is the case for the open question of consciousness and subjective experience. Weak emergence can be usefully defined through computational irreducibility (Bedau, 1997). An advantage of this definition is that it may be used for any process that carries out computations (as is the case for optimization on models). An emergent behaviour may not be reducible to the behaviours of the independent parts (Holland, 1992) (see Figs. 1 and 5).

*Computational Irreducibility* A computational process which has no shortcut. The only way to achieve the end result of the computational process is to run the entire computational process (Bedau, 1997; Zwirn & Delahaye, 2013).

*Phase Space* The underlying spaces (or fields) in which phenomena and models exist (Longo, 2018). Some claim strong emergence to be a property of complex biological ecosystems, or complex social systems like economics, whereby the phase spaces of these systems undergo changes and transformations which can not be reduced to computational steps, or sequences of an optimizaiton (Bailly & Longo, 2009).

*Open* A system which does not have precisely defined boundaries: a system where external variables that are not accounted for influence the evolution of the system (Von Bertalanffy, 1950; Chick & Dow, 2005) (see Figs. 1 and 4).

### The Limitations

**Fig. 2:**
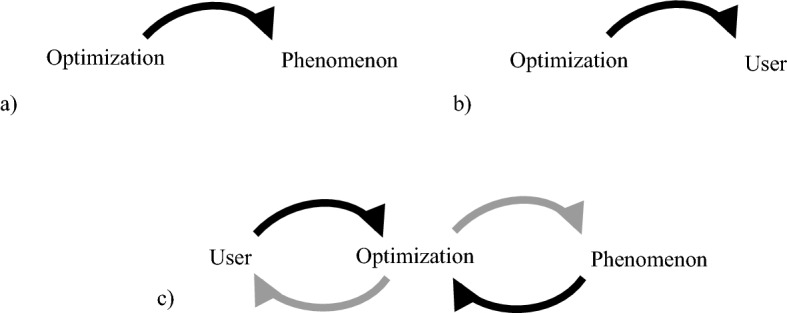
Limits to optimization come in three categories: **a** the relation between optimization and the phenomenon through the process of modelling, **b** the relation between optimization and the user through the process of choosing an optimality criterion, **c** the feedback from the phenomenon and the user which can affect the model and the optimality criterion

There are three main limitations stemming from the above definition (vizualised in Fig. 2):

- Object limit (Sect. 3): pertains to the relation between optimization and the phenomenon, which is facilitated by the model *M*.
- Objective limit (Sect. 4): refers to the relation between optimization and the user, encapsulated by the choice of the optimality criterion *f*.
- Process limit (Sect. 5): when the process of optimizing itself affects either the model *M* or the optimality criterion *f*. That is, by selecting objects *m* from *M* or by applying *f* to *m* the user affects the phenomenon, which in turn may affect the model and/or notion of optimality.

The first limit is mostly an epistemic one, we cannot optimize something we cannot model. Greater computational resources have allowed us to extend the phenomena we appear to model accurately, but it remains an open question (partially addressed in this paper) whether it is possible to perfectly model all phenomena. The second limit focuses on the meta-problem of selecting an objective, that is answering the question: *What are we looking for?* The third limit is an enactive one, where we consider the process of optimization to be embedded in the phenomenological sphere and thus being able in particular cases to influence its own functioning.

Our endeavor presently will consist in explaining these three limits, providing examples and lingering on the questions infer-able from them.

## Object Limit

The objects of an optimization are the quantities *m* used to construct a quantitative model *M* of a phenomenon. Most models are not in a one-to-one relation to what they model and so any approximations we take here might translate into inaccuracies in the final results of the optimization process itself. This inaccuracy of models has been addressed in depth by the field of General Semantics (Korzybski, 1958) which focuses on the language as a modelling tool and its many inadequacies with respect to representation and understanding. Thus, limits that pertain to the objects are representational limits. We identify two such limiting factors, corresponding to the first two rules of General Semantics^3^.

1. A model *M* may be inaccurate with respect to the phenomenon it models, such that it does not capture the true behaviour of the phenomenon in all circumstances.
2. The phenomenon being modelled may be complex, have emergent properties and be computationally irreducible requiring a model to be nearly as expressive as the entire phenomenon, such that modelling actually introduces greater complexity than it provides simple understanding.

Physics is the field of science recognized as having the most accurate models of phenomena (Oerter, 2006). For example, our models of particles and electrons are so accurate [matching measurements in experiments up to 12 decimal places (Sailer et al., 2022)] that we can construct computers. Computers are capable of carrying out logical operations that are not described by the models of their constituent particles and electrons. Yet, these models were necessary to create computers, and the fact that their operations transcend these models is a testament to the practical use of the models.

Models of social phenomena, on the other hand, tend to have lower accuracy than models in physics (Beckage et al., 2013), as in studies of life success given IQ, for example, where correlations above 0.5 are taken to be indications of strong relationships (Firkowska-Mankiewicz, 2002). This is accepted by the psychologists and social scientists who recognize the inevitable challenge of modelling social phenomena: there is great heterogeneity between human subjects (Axelrod, 1997) as social systems are open systems affected by many factors that we cannot account for (Green & Perlman, 1985). This, however, does not stop users from applying optimization techniques on inaccurate models of social systems [as in the optimization of click-through-rates (Richardson et al., 2007) and content recommendations (Cremonesi et al., 2010) on websites], because the mathematics of the optimization techniques does not break down. What is limited is the applicability of the results of the optimization to a real understanding of the modelled social system.

**Fig. 3:**
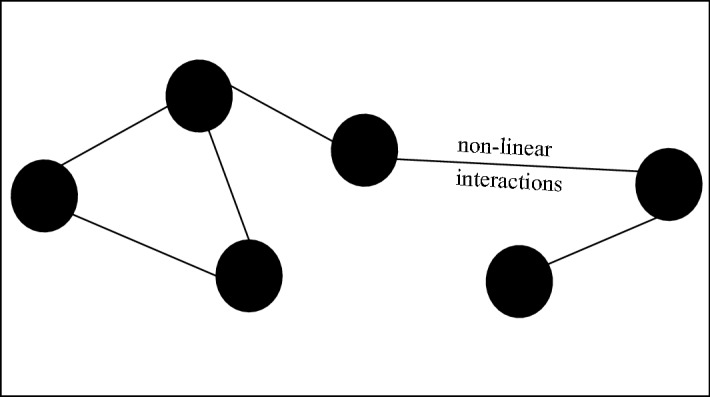
Vizualisation of a complex system with non-linear interactions between the parts

**Fig. 4:**
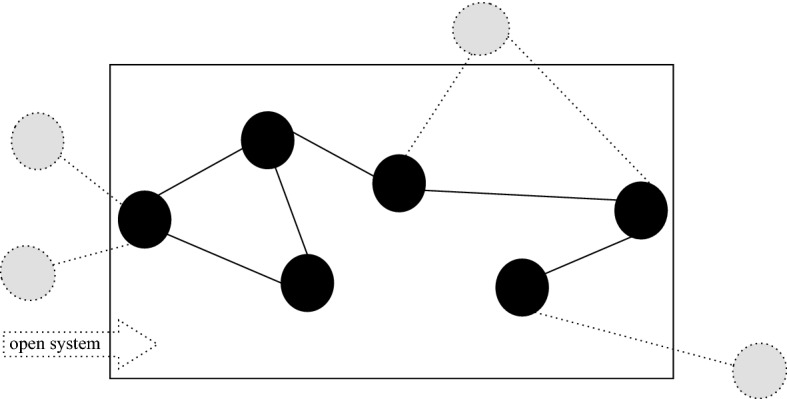
Vizualisation of an open system where parts that are not within the model closure interact with the parts within the model closure

**Fig. 5:**
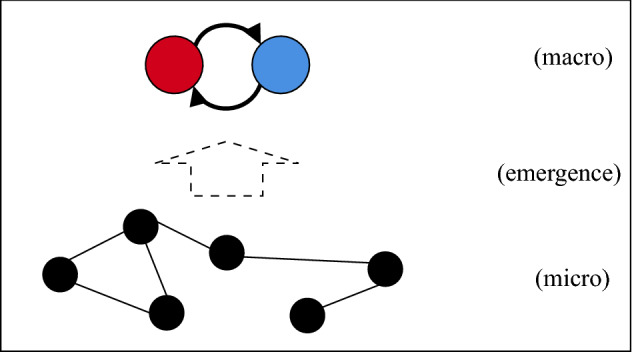
Vizualisation of a complex system with an emergent collective behaviour. The emergence results from the local interactions at the micro scale which lead to a collective behaviour at the macro scale

An example of a whole class of systems for which such limits may apply are complex systems, whose elements and interactions generate novel order that cannot be defined a priori (Batty & Torrens, 2001) (see Fig. 3). Then open systems, which are under influence of external driving forces, can also prove difficult to model accurately, depending on the nature and degree of openness that they exhibit^4^ (Von Bertalanffy, 1950) (see Fig. 4). When the interactions between systems is such that the phase spaces change, it may be impossible to quantify the possible future from a model which is merely a snapshot of the past (Longo, 2018). This may be a hard limit of modelling and controlling complex systems that are strongly dependent on their histories, such that the present state of the systems (or that which is feasibly measurable) does not contain the sufficient information to determine all future possibilities in interaction with other complex systems. Similarly, weakly emergent systems cannot be fully modelled a priori, because their macrostates can be derived from their microstates only through simulation (in other words they are computationally irreducible) (Bedau, 1997) (see Fig. 5).

Moreover, recent developments in machine learning have led to many models being learned directly from data, to score the credit worthy-ness of people for example (Ala’raj et al., 2022). The process of generating these models, where deep learning is employed, can itself be considered an optimization process (Bennett & Parrado-Hernández, 2006). Optimization methods play a central role in the ability of machine learning algorithms to extract information from data. These models are naturally limited: they are only, at best, as good as the data. The usual procedure when optimizing such models would be to decouple the model learning from decision making—machine learning creates the model from the data and then some algorithm optimizes, treating the learned model as if it were exact. In Balkanski et al. (2017) the authors show that there exist classes of functions, which are not optimizable in this way. This limitation stems from sample complexity rather than from computational complexity. In other words, there are situations in which the data does not carry sufficient information to allow optimization.

We have echoed an often claimed notion that models are not perfectly accurate, and provided some examples to substantiate it. However, discussing optimization as being limited by models runs into ontological questions of the existence of the phenomena. Should one take a computationalist perspective, whereby any phenomenon *P* is in its most fundamental essence a composition of computational processes, then it naturally follows that there exists a computational model *M* such that the phenomenon is fully captured by the model, $M=P$; in other words, that there exists a perfectly accurate model.

Let us assume that this computationalist perspective holds, and consider the case where we run an optimization on top of our model. For the optimization to offer practical benefits it must at some point return a global or local, an exact or approximate optimum, that is the process must necessarily be finite. Thus, a fixed optimization horizon N must be established that delimits the time allocated for optimization. Therefore, it is important that the model includes all the relevant behaviour for the modelled phenomenon within the optimization horizon. N is picked such that all of the behaviours we wish to model have sufficient time to occur within N iterations.

However, if the system is computationally irreducible, there is no guarantee that all interesting phenomena will occur within N iterations: ‘if the behavior of an object is computationally irreducible, no computation of its *n*th state can be faster than the simulation itself’ (Zwirn & Delahaye, 2013). Should this definition of a computationally irreducible process hold, a computationally irreducible system may thus have behaviour *b* for which a fixed time horizon *N* can not be stated in order for *b* to manifest with complete certainty. Therefore, optimizing a model *M* of such a system (in the manner specified in Sect. 2) may yield an *m* which is not optimal on the unobserved behaviour *b*. Claiming such an *m* to be optimal in all cases is therefore a fallacy.

If the iteration horizon is a boundary on time, set such that the model of our phenomenon is accurately represented, the model horizon is a boundary on interactions and causal influences, to close an open system off from the external world. The model horizon is another limit of optimization particularly heightened for complex and open systems. For a complex, open phenomenon there may be unknown and uncountably many external variables, relevant to the phenomenon and yet excluded from the model such that a tractable model of the system could be designed (Chick & Dow, 2005). The effects of the variables falling outside of the boundary (outside a Markov blanket for example), though relevant, become irrelevant to an optimization run on the model. Again, from a computationalist perspective, the inaccuracy of the model is simply an epistemic factor, which could in principle be complemented by greater processing power to achieve a complete and thus perfectly accurate model.

However, taking a non-computationalist perspective, there exist phenomena which can not be modelled completely, because phenomena are not, in their essence, fully reducible to input output relations, computations. Although computationalism may hold, science so far has not produced perfectly accurate models, as was argued in the previous paragraphs mentioning physics and social models.

## Objective Limit

Assume we have selected an appropriate model *M* to embody the phenomenon *P* we are studying and that this phenomenon is indeed satisfying all the assumptions that optimization imposes. A meta-problem of deciding what to optimize is the next logical step of optimization. Indeed, optimization necessitates the existence of an explicit goal (expressed by the valuation *f*), thus to optimize is to focus on the aims rather than the means. The aim must be defined, we must be able to discern it clearly to know when it has been reached. This meta-problem might itself be optimized, resulting in an infinite regress (Hubinger et al., 2019). Beyond the model, one should not appeal to the notion of optimum, but rather of choice. The optimality does not provide information on the choice the user ultimately makes. The decision to be made is a value choice, namely the user must decide what value *f*(*m*) of the phenomenon (expressed through the model) will be the goal of the optimization. At our current stage of sociotechnoligcal development, the decision is made by humans. One could of course imagine this being offloaded to an oracle or randomness or in some futuristic scenarios to AGI.^5^

The selected goal needs to be quantifiable. We need to be able to compare alternative states and decide which one of them is better. Some values, like trust, friendship and love may not always be quantified and readily optimized, unless modelled dependent on quantifiable values. Thus, optimization imposes further constraints on the way a system is modeled, by requiring the system’s states to be measurable with the objective. If we want to be able to optimize, we risk preferring models that allow for optimization [which may be a reason we observe a strong preference of quantitative models in modern science, as is especially apparent in e.g. economics (Bruni, 2010; Mirowski, 1991; Romer, 2016)]. In that way we can end up framing and understanding the system through the perceived goal that we have for it—instrumentalizing it and potentially limiting our perspective.

**Fig. 6:**
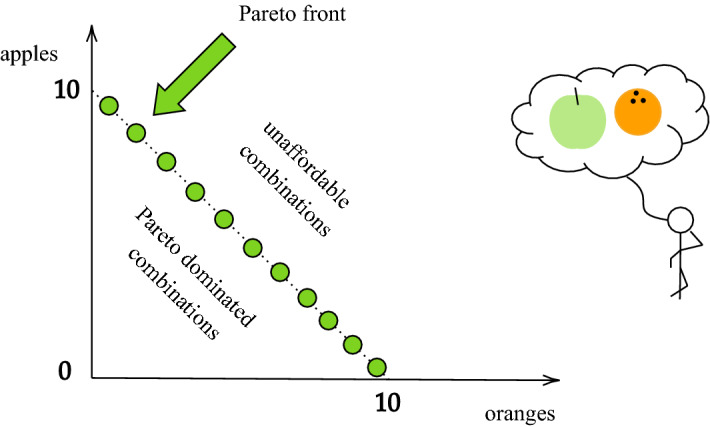
Imagine a customer at a market stand that sells both apples and oranges. Our customer has grown up with the notion that apples and oranges should not be compared, so he does not prefer either fruit more than the other. Ideally, he would like some combination of both. With his pocket money, he can afford different combinations of apples and oranges (apples, oranges) e.g. (10, 0), (5, 5), (1, 9). As he does not compare apples and oranges, he is indifferent between all the combinations that get him the most possible apples and oranges. That means that all of the combinations are optimal in the same manner. Our customer therefore must resort to other means to decide which of the combinations to pay for and take home. We say that the set of combinations that our customer is indifferent to is the Pareto front, where all the combinations are optimal under a definition of Pareto optimality (Deb, 2014). A combination is Pareto optimal if no improvement can be made on the combination such that we get more of one fruit without getting less of the other fruit. For example, the combination (4, 5) in this example is not optimal, because the combination (5, 5) is affordable and gets our customer one more apple

**Fig. 7:**
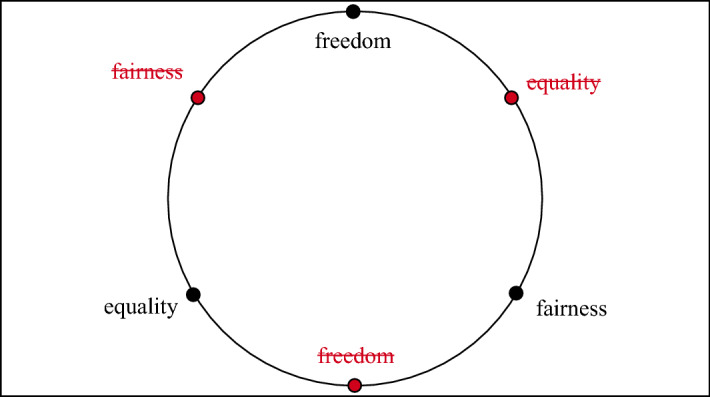
According to Steven Pinker (2005a, 2005b) our western societies wish to provide freedom, equality and fairness, which he argues to be a trilemma. This implies that only two of the three desirable options can be achieved fully at the same time: society can be fair and free, fair and equal or equal and free. For the figure, possible states lie on the boundary of the circle. Values are maximized at the black points, and are 0 at the opposite side

Secondly, there is an issue with the number of values that can be optimized. In simple optimization problems we select one goal and optimize with respect to it. However, in many real-world problems there is a set of goals that we want to achieve. This necessitates a new notion of optimality, an example of which is Pareto Optimality (Deb, 2014). Figure 6 explains Pareto optimality in greater detail with the use of an example: comparing apples and oranges. Figure 7 provides another example of a challenging multi-objective optimization: Steven Pinker’s social trilemma of fairness, freedom and equality (Pinker, 2005a, 2005b). The challenge introduced by multiple objectives is that optimization does not provide an answer to which of the objectives should be preferred. The optimization does not terminate and yield a single solution that can be followed. If the user is a non-normative being, then they will not be able to choose, since the optimality does not provide the necessary information to choose. Choosing is a moral process, not a computational one. It is an *is-ought* fallacy to derive what the user should choose based on the Pareto front.

Focusing on the user, who we have assumed to be human we realize that they are befallen by an epistemic limitation—*What does one know?* (Williamson, 2002)—one half of the question—*how can we find something that we do not know we are looking for*. In some systems one might conceivably know what needs to be optimized and be able to reason about it analytically (abstract systems, simple systems). In other systems there is a strong argument against assuming too much knowledge. For example, in many complex systems (traffic, the economy) we might not have a full understanding of how a change in one variable of the system might affect the whole system (Ladyman et al., 2013). In such systems, optimizing a given variable might lead to a feedback loop, which eventually drives the optimized variable to a new state which may invalidate the optimization (McDaniel & Driebe, 2005).

Stemming from the fields of evolutionary algorithms and machine learning is also a new awareness that objectives are limited in scope (Lehman & Stanley, 2011). Kenneth Stanley in a recent book (Stanley & Lehman, 2015) argues that for ambitious objectives, following the objective typically leads to dead ends. He claims that using the objective as a measure of improvement, and only taking actions that improve along this measure, is unlikely to lead to the objective, when the latter is ambitious. An ambitious objective is conceptualized as one that requires a sequence of novel advancements that are not known beforehand, an example of which could be writing a ground-breaking scientific paper, or leading a satisfying life. A practical example from evolutionary optimization is teaching a robot to walk. When the robot tries to improve the distance it travels, falling face first at the first step counts as an improvement of distance, but is clearly not a step in the right direction. Along this objective measure of distance, bending the robots legs does not constitute an improvement, but is a necessary thing to learn to be able to achieve a steady walking gait. Such examples are used to argue that, for ambitious objectives, the objective gradients should not be followed.

Stanley proposes instead that a gradient of ‘interesting-ness’ should be followed. We refrain from agreeing to this as a complete solution, but note that interesting-ness itself is a highly subjective quality. This is because, what is interesting has historical dependence, meaning that *A* may only be interesting given the experience of *B*, as well as a dependence on the outcome, since *A* may be interesting given that it leads to *B* (given *A* and *B* as arbitrary phenomena). This makes quantifying interesting-ness an ‘interesting’ challenge. One proxy quantification of interesting-ness proposed by Stanley is novelty, where Stanley argues that anything interesting will be novel, while not everything novel will necessarily be interesting. It is ‘interesting’ to consider how path dependent phenomena such as interesting-ness interact with optimization, where it could be argued that historical dependence leads to an inherent unpredictability, as has been done for historicity in cognition and biology (Longo, 2018), and this could create a further epistemic limit for the modelling of the underlying phenomenon. This kind of feedback is central to the ‘Process Limit’ we discuss in Sect. 5.

## Process Limit

This is perhaps the least intuitive of the limits, while also being the most socially relevant one. Optimizations themselves may affect the phenomenon and its model, both being optimized, in such a way that the system under study changes considerably. Given our definition of optimization, the process may affect either *M* or *f*.

1. If it affects *M*, then the objects that we use to represent our system or the system itself may change. Our optimization may become obsolete if the changes to these objects are considerable.
2. It if affects *f*, then the values that we assign to object manipulations may change, such that by optimizing for the original values, our optimization may actually be detrimentally affecting our new value.

One way to understand these cases might be to frame them as instances of learning, that is to see optimization as an epistemic process. As optimization is performed it is conceivable that we learn something new about the phenomenon, its model or our valuations.

On the other hand, optimization can also be seen, in some cases, as interfering with the phenomenon. Depending on what kind of system we are optimizing, the application of valuation *f* to a given object $m \in M$ can affect the object *m* itself or the other objects of set *M*. This problem will not occur for abstract systems. When we want to find a minimum of a function, no matter how we traverse it we will not affect any of its objects. For embodied and embedded phenomena, however, it is conceivable that by interacting with them we might affect them.

**Fig. 8:**
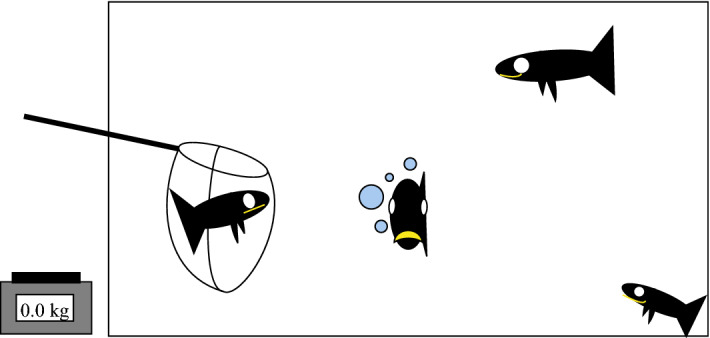
Imagine we have a huge aquarium full of different species of fish with similar weights. Our goal is to find the heaviest fish in the aquarium by weighing the fish on a scale. When we take out the fish one by one to weigh them they may react differently. Some of them swim agitated, others defecate profusely and the remaining ones greedily stress-eat. These minute behaviours can have an influence on their weights (!) and this optimization process is interfering with the closed system of the aquarium

Figure 8 provides a simple example that captures such an effect: when measuring the weights of fish, this may affect their weights. In this example, the pursuit of an objective requires a measurement which may have a non-trivial effect on the system being measured. In other words, it is a case where a measurement is not ‘free’ or independent of the system being measured. In fact, it will often be the case that interacting with a complex system (like a fish, any other biological entity, or a system of such entities) has a cost. This cost could be energetic, pertaining to an effort that is required to take a measurement, and it could be that this cost is born by both the measurement taker and the measurement subject (as in the case with the fish). If any cost is also borne by the subject, it may be challenging to predict how this effect will affect future measurements. The measurements need not affect the phase space of the underlying system for this limit to be relevant, as indeed the phase space of the aquarium has not changed following measurements since the behaviour that leads to alterations of the fish weights is due to the same biological responses and behaviours that the fish could display before the measurements (swimming, digestion, eating).

A similar effect occurs in many social situations. Consider the SATs exams as an optimization process, whose aim is to determine the best students. The exam can be considered the valuation *f* and the students are elements of the set *M*. By applying the valuation, we are clearly affecting the students. They are made aware of the exam and now prepare explicitly for it or resort to cheating instead of just being good students (Klein et al., 2000). This is an example of the optimization process affecting the system it optimizes and driving it into states which were not intended. This example differs from the fish example (Fig. 8), as the process of measuring SAT scores not only influences the scores it also creates a new adaptive niches for the students: for example the possibility to study to the test format, and the possibility to cheat the test. Thus the optimization has affected the underlying phase space of the system, and not just the performative ability of the students.

Following up on that example, one could imagine this process leading to inequalities, where not necessarily the best students will achieve best SAT scores but rather ones that due to their material conditions had access to tutoring and SAT preparation courses (Boaler, 2003). Once this realisation is made, the valuation *f* changes as it no longer optimizes only for the skills of the student but also for their wealth.

These effects could be considered a form of Observer’s Paradox (Labov, 1972) whereby the fact that there is an observer optimizing a system unwittingly affects the system being optimized. A common example of the Observers Paradox is the ‘Hawthorne Effect’, dubbed as such following the peculiar behaviour of factory workers during a study conducted between 1924 and 1932. A firm had commissioned a study to understand how much lighting was optimal for the workers. To their surprise, the workers performance increased every time the lighting was changed: more light, less light, and back to the same amount of light. A popular explanation provided by Landsberger (1958) is that the workers were performing better because they were being observed. In fact, after the study, their performance slumped.

A further example of the valuation *f* being affected by the process of optimization is explained in detail in Hubinger et al. (2019), where mesa-optimization is introduced. Mesa-optimization is a case where a learned model is itself an optimization. In such a scenario, the inner-optimizer may have an internal objective function $f^i$ which is mis-aligned with the external objective function $f^e$.

This concludes our explanations and examples of the three limits of optimization due to objects, objectives and the process. In the next section we bring these limits together to spur and stimulate a thoughtful discussion.

## Discussion

In this paper we have defined optimization as the process of finding the best object in a set of objects for a particular optimality criterion, and argued that it can be limited in three main ways: objects, objectives and the process. While there may be overlap between these categories, we find it helpful to conceptualize their differences, and examples are provided for each of these limits. This endeavour raises several questions, which remain open and for further investigation.

### What are the Alternatives to Optimization?

We have claimed that while optimisation is a tool that can be applied in some settings, it is not appropriate in others. For the clarity of the argument it appears fair to discuss if there are any alternatives to optimisation. Since, optimisation is widely used (even in settings where it perhaps should not be) it is natural to attempt to offer a different approach that could perhaps avoid the limits inherent in optimisation, while achieving the goals that are intended by it.

One process that has continuously served as an inspiration in many disciplines of science is evolution. Evolution and its natural selection has been claimed to be significantly different and more complex than a simple optimisation. From the perspective of our arguments, one key difference between evolution and optimisation is that the latter is expected to be a finite process yielding a final result, while evolution is a continuous process without a clear end or goal. Furthermore, the results of natural selection can hardly be considered optimal.^6^ Moreover, the underlying phase space of the evolutionary process is a priori unmeasurable (Bailly & Longo, 2009) and so establishing an order over it, which would be necessary for optimisation to occur, is impossible.

Similarly, economic opportunity does not fit uniquely into an optimization paradigm. Parallels are drawn between biological evolution and the evolution of economic opportunity, such that it is not sufficient to conceptualize a static landscape of opportunity, but a dynamic and history dependent one (Felin et al., 2014). Thus the phase space of economic opportunity is modified and changed by the economy itself (Koppl et al., 2015), which undoubtedly contains some optimization processes, but is not itself an optimization process; since optimization alone is not sufficient to explain the emergence of economic opportunity.

Thus, it appears, evolution and economy would be some of the examples of a process that could be free of many of the limits of optimisation, while at the same time still leading to results, which can a posteriori be considered desirable in some sense. Here it is worth noting that the evolutionary algorithms, used in computational science, while inspired by evolution, are still essentially optimisation (fitness function requires ordering). Perhaps, the understanding of evolution and economy referenced here could inspire further methodologies (including computational methods) that would be distinct from optimisation and free of its limits.

### Are Humans Reducible to Optimization?

Is every thought and action we take conditioned and in service of some explicit optimum that we aim to achieve? Need we be reduced to continuous quantification, modelling and comparison of alternatives?

From a teleological perspective, human actions and thoughts can be understood and explained in terms of their purpose. This, however, is not the same as reducing them to optimization. While the effects of some human actions might be optimal with respect to some criterion, they are not necessarily the result of optimization. The fact that humans take actions that have purposes and outcomes is not enough to reduce human actions to optimal behaviour. The fact that an action can be considered optimal does not imply that it has resulted from an optimization process. In fact the optimality of the actions can only be asserted a posteriori by relating them to an objective that is external and separate to the action or its goal.

This teleological perspective does not preclude some human actions from being optimal, and there are human behaviours, which appear to incorporate optimal value based decisions. Neuroscience, for example, has successfully modeled and described several such optimal behaviours in the realm of perception and attention (Tajima et al., 2016; Brus et al., 2021). Nonetheless, as argued above, the fact that these behaviours lead to optimal outcomes cannot be used to infer them as results of an optimization.

**Fig. 9:**
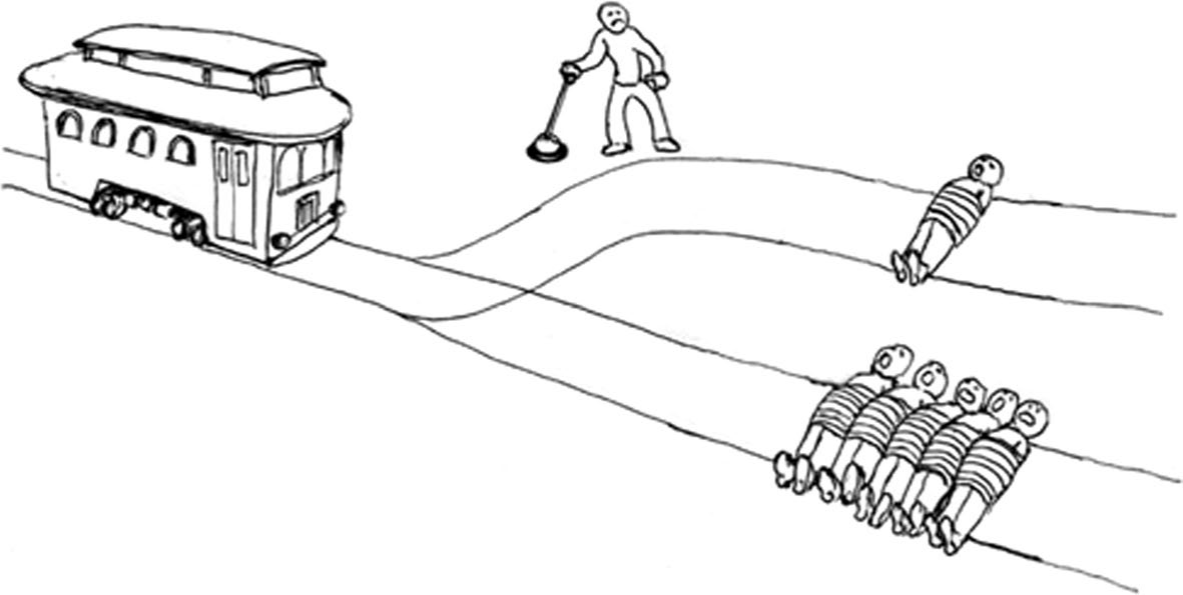
The Trolley Optimization (?): optimization could be used to determine which choice is best according to an objective function, but requires a choice of objective to do so. The trolley problem, for example, could be optimized using the number of people harmed, but it is a normative decision to use such a utilitarian objective. The fact that it is called the trolley ‘problem’ (and not the trolley ‘optimization’) could be an indication that most reasonable individuals do not believe there to be an optimal decision

Another limitation of reducing human actions to optimization is that optimization does not offer a normative dimension, that is it does not in any way contribute to answering the question *what decision should be made?* Therefore, if one believes humans do make normative decisions, then no more can be learned about how that occurs using optimisation. Indeed, from a computational perspective any normative decision could be reduced to optimisation. On the other hand, if we do not subscribe to computationalism, optimisation does not allow us to study the normative decision making process further. Figure 9,^7^ presents a common sense argument for the existence of normative decisions.

This raises an interesting perspective on artificial intelligence research. If we consider human intelligence to be irreducible to optimization it becomes unlikely that a human-like intelligence can be achieved with just optimization techniques. How is the AI supposed to make decisions if it can only optimize (Weizenbaum, 1976)? Nevertheless, currently, one of the most driven branches of AI, including machine learning and especially deep learning, focus almost exclusively on optimization. In fact, deep neural networks are just parametric models optimized with stochastic gradient descent. As such they are constrained by the same set of limitations that we have delineated in this paper. Therefore, the limits of optimization in machine and deep learning should be of great interest to philosophers.

### Should Societies be Universally Optimized?

We have argued that it is beyond our ability to model society with perfect accuracy given that it is an open and complex system. To overcome this limitation, we may try to model and optimize reduced parts of the the social phenomenon rather than the whole. But as we have indicated, complex systems are not generally reducible to their parts. Optimizing one part of a system (eg. the socioeconomic situation of the top 10%), without considering the interactions that part has with the other parts (eg. the socioeconomic situation of the bottom 90%), can lead to emergent phenomena that are challenging to foresee and that cannot be understood with the model of just one of the parts.

Moreover, the meta-problem of selecting the values that need to be optimized is more challenging for a society. This is because society is already an aggregate of many individuals. When these individuals each have different needs, desires and goals, how should a unique optimization criterion be formulated? Picking the goal that should be pursued is a normative decision which an optimization does not provide. If we attempt instead to satisfy all goals, the optimization will not yield a unique solution, but rather a set of solutions. Picking from this set of solutions, once again, is a normative decision, which the optimization does not provide. Does there even exist in principle, a goal that all individuals pursue, and that a society can optimize for?

Profit is often taken to be the goal that all individuals pursue, and that a society can optimize for globally. It is so appealing because it is easily quantifiable, and all actions can be compared in terms of their effects on profit. Behaviours like spending and consumption, which clearly have societal impacts are aggregated *en masse* for individuals and companies and used to estimate Gross Domestic Product (GDP). Changes in GDP are then used to justify interventions in society. Nations optimize their politics and laws while optimizing for GDP.^8^ Following our arguments for the limits of optimization in open and complex social systems, do nations of today have a sound model and goal for societies?

What about emotions? What about moral behaviour? Can this be quantified? It is questionable whether human experiences like emotions or morality can be quantified in principle, and it is also questionable whether it is ethical to try. We recall a powerful phrase by Robert F. Kennedy addressing The University of Kansas on March 18th 1968: ‘[GDP] measures neither our wit nor our courage, neither our wisdom nor our learning, neither our compassion nor our devotion to our country, it measures everything in short, except that which makes life worthwhile. And it can tell us everything about America except why we are proud that we are Americans’. What directions does a society go in, when guided by actions that optimize for ‘everything, except that which makes life worthwhile’ (GDP)?

Again, we draw an interesting link to artificial intelligence and societies. Most people will have entertained the idea that perhaps, large-scale social problems like climate change, global pandemics, systemic inequalities, and the resurgence of totalitarianism could be ‘off-loaded’ to an artificial general intelligence (AGI). This machine would be so much more powerful than any individual, and capable of bringing together many objectives to find the forward path that solves them all simultaneously. Based on our arguments, such an idea should be entertained with great care. The AGI would be acting and influencing the society it is trying to optimize using a model that humans cannot interpret. The AGI’s model may be more accurate than human models, but it will nonetheless be subject to object, objective and process limits. Furthermore, optimization does not provide the tools to decide what is important. Such decisions are made by the users, and it seems reasonable for those decisions to be taken by the users most affected by the consequences. Putting an AGI in charge resembles putting an optimization process in charge.

### Final Remarks

We have defined optimization and discussed the limitations pertaining to optimizing models of real phenomena, particularly for open and complex social systems (Sect. 2). We characterized these limitations with toy and real examples (Sects. 3, 4, 5). We concluded with an open discussion on the ways that optimization shapes our understanding of individuals and societies (Sect. 6). Before us lies extensive future research. Practically, we aim to further the collection of examples that clarify the limits of optimization. Theoretically, there may be valuable results that can quantify the limits of optimization for open complex systems, provided sufficiently descriptive definitions of openness and/or complexity.

## Footnotes

1 Similar to the question: ‘How can we look for something that we do not know yet?’ (Hayek, 2002). Hayek explains how competition in the free market discovers new facts that cannot be predicted since they depend upon the agents’ arbitrations—which, in turn, are free hence undetermined.

2 The authors note that computationalism as defined here contains more ontological implications that are often debated in the philosophy of mind: the structure of physical systems, their informational content and their compositional properties. The aforementioned computationalism subsumes the weaker, epistemic, philosophy of mind version, that explain cognition and consciousness as computations carried out by physical processes in the body and brain.

3 The first two rules of Korzybski’s General Semantics are:

1. A map is not the territory.
2. A map covers not all the territory (Korzybski, 1951).

4 ‘Verification and validation of numerical models of natural systems is impossible. This is because natural systems are never closed and because model results are always nonunique.’ (Oreskes et al., 1994)

5 Even so, off-loading the decision in such way could still be interpreted as a human making a decision (to off-load the decision, at the very least) with all its trappings and consequences. The delegating does not clearly free the delegator of the responsibility or the value-choice; it could be considered a sort of meta-decision.

6 ‘The result is far from optimal, even less an optimum, at least because there is no pre-given partial order, where a largest element could be defined: the ecosystem is co-constituted by the dynamics and no pre-given choice of (mostly incomparable) observables may allow to fix a partially ordered space with a maximum or even local maxima. Indeed, if an organ or an organism were an optimum in a given phase space, and this optimality were a (essential) component of its fitness, as claimed by the Modern Synthesis, it would be very soon dead, as the ecosystem changes.’ (Longo, 2018)

7 The image has been reproduced as an Internet meme, retrieved from http://subcortex.com/pictures/, personal website of prof. Jesse Prinz believed to be the original author.

8 GDP is used as an illustrative example and does not imply any systematic statements. Microeconomics of the Neoclassical school as well as the Austrian school reject the macroeconomic notion of GDP as meaningful or having any bearing on political actions that should be taken. Such a rejection is in fact convergent to a large degree with the arguments presented in this paper.
